# Potential effectiveness of Chinese herbal medicine *Yu ping feng san* for adult allergic rhinitis: a systematic review and meta-analysis of randomized controlled trials

**DOI:** 10.1186/s12906-017-1988-5

**Published:** 2017-11-06

**Authors:** Qiulan Luo, Claire Shuiqing Zhang, Lihong Yang, Anthony Lin Zhang, Xinfeng Guo, Charlie Changli Xue, Chuanjian Lu

**Affiliations:** 1 The Second Affiliated Hospital of Guangzhou University of Chinese Medicine, 111 Dade Road, Yuexiu District, Guangzhou, 510120 China; 2 Guangdong Provincial Hospital of Chinese Medicine, 111 Dade Road, Yuexiu District, Guangzhou, 510120 China; 3 The Second Clinical College of Guangzhou University of Chinese Medicine, 111 Dade Road, Yuexiu District, Guangzhou, 510120 China; 4 China-Australia International Research Centre for Chinese Medicine, School of Health and Biomedical Sciences, RMIT University, Bundoora, VIC 3083 Australia

**Keywords:** *Yu ping feng san*, Chinese herbal medicine, Allergic rhinitis, Treatment duration, Systematic review, Meta-analysis

## Abstract

**Background:**

Chinese herbal medicine formula *Yu ping feng san* (YPFS) is commonly used for allergic rhinitis (AR). Previous review had summarized the effectiveness and safety of YPFS, however without any subgroup analysis performed to provide detailed evidence for guiding clinical practice. YPFS was recommended for the management of AR by Chinese medicine clinical practice guideline, but the treatment duration of YPFS was also not specified. The aim of this study is to evaluate the effectiveness and safety of YPFS in treating adult AR with the most recent evidence, and attempt to specify the duration of utilisation through subgroup meta-analyses.

**Methods:**

Seven databases were searched from their inceptions to September 2017. Randomized controlled trials (RCTs) evaluating YPFS for adult AR were included. Methodological quality of studies was assessed using the Cochrane risk of bias tool. Meta-analysis and subgroup meta-analyses were conducted for evaluating the effectiveness of YPFS. The Grading of Recommendations Assessment, Development and Evaluation (GRADE) approach was used for rating the quality of evidence.

**Results:**

Twenty-two RCTs involving 23 comparisons were included in this review. YPFS was compared to placebo, pharmacotherapy, and used as an add-on treatment compared to pharmacotherapy. Meta-analyses were feasible for the outcomes of four individual nasal symptom scores and “effective rate”. Four individual nasal symptom scores decreased after YPFS’ combination treatment: itchy nose (MD-0.46, 95% CI[−0.50, −0.42]), sneezing (MD-0.41, 95% CI[−0.47, −0.35]), blocked nose (MD-0.46, 95% CI[−0.54, −0.39]) and runny nose (MD-0.42, 95% CI[−0.58, −0.26]). Based on “effective rate”, meta-analysis showed that YPFS did not achieve better effect than pharmacotherapy (RR1.07, 95%CI [0.94, 1.22), but its combination with pharmacotherapy seemed more effective than pharmacotherapy alone (RR1.27, 95%CI [1.19, 1.34]) (low quality). Subgroup analysis suggested that YPFS was not superior to the second-generation antihistamine (RR1.04, 95%CI [0.90, 1.19]) (low quality). Further, YPFS’ combination treatment seemed more beneficial when it was used for more than three weeks (RR1.15, 95%CI [1.01, 1.32]). In addition, YPFS was well-tolerated for treating adult AR.

**Conclusion:**

Chinese herbal medicine formula YPFS seems beneficial for adult AR. This potential benefit need to be further evaluated by more rigorous RCTs.

**Electronic supplementary material:**

The online version of this article (10.1186/s12906-017-1988-5) contains supplementary material, which is available to authorized users.

## Background

Allergic rhinitis (AR) is the most common type of non-infectious rhinitis; it is defined as a “symptomatic disorder of the nose induced by the IgE-mediated inflammation after allergen exposure” [1]. AR is characterized mainly by nasal symptoms including sneezing, itching, rhinorrhoea and nasal congestion, as well as a group of non-nasal symptoms involving eyes, ears, throat or chest. AR patients may also develop sinusitis and asthma, or be accompanied by malaise, weakness, and fatigue [2, 3]. Globally, AR affects 10% to 20% of the population, and an increase of the prevalence has been observed in the past 40 years [1, 4]. Clinically, AR is one of the most common diseases affecting adults [5], and it is considered a major chronic respiratory disease due to its high prevalence, impairments on patients’ quality of life (QoL) and work/school performance, substantial economic impact and its co-morbidities [1, 6].

Allergic rhinitis has traditionally been categorized as seasonal or perennial types according to the predilection time and triggering allergens, or may be classified as intermittent or persistent types based on its frequency and severity. However, both of these classifications have certain limitations [1, 5–7]. Regardless of the classification types, their clinical management are all same. Recommended by the most recent clinical guideline, effective treatments of AR are topical steroids, oral antihistamines, and immunotherapy [5]. This guideline also pointed out that Chinese herbal medicine (CHM) has often been utilized for managing AR in clinical practice, although the evidence supporting its efficacy and mechanism of action is uncertain [5]. As suggested by a systematic review published in 2012, CHM might be more effective than placebo for persistent allergic rhinitis, but a confirmed conclusion could not be drawn since all included studies suffered certain methodological limitations [8].One CHM formula, *Yu ping feng san* (YPFS), was recommended by Chinese medicine clinical practice guideline to manage AR despite its classification and severity, but the effective treatment duration is not clear [9]. YPFS formula contains three key herbs: *Astragalus membranaceus* (*huang qi*), *Rhizoma Atractylodis Macrocephalae* (*bai zhu*) and *Radix Ledebouriellae Divaricatae* (*fang feng*) [10]. In clinical practice, YPFS usually is used together with other herbs, e.g., Lilymagnolia (*xin yi*), Fructus xanthii (*cang er zi*) and Radix Angelicae Dahuricae (*bai zhi*) etc. The effectiveness and safety of YPFS for AR treatment has been investigated by a number of clinical studies, and been summarized by a review article published in Chinese [11]. Although the overall effectiveness of YPFS seems promising, the treatment details were not suggested due to the lack of subgroup analyses. Therefore, in depth meta-analyses of current evidence are needed for guiding the use of YPFS for adult AR treatment in clinical practice.

## Methods

The review protocol was registered on PROSPERO (CRD42015024821), and the review was constructed following the PRISMA guidelines (Additional file 1).

### Study selection criteria

This systematic review was designed to evaluate the effectiveness and safety of CHM formula YPFS for AR. No limits on language and publication type were placed on study selection. Studies were considered if they recruited adult AR participants (aged 18 years and above) without classification limited. Included studies were RCTs comparing oral YPFS in any preparation forms to placebo CHM or pharmacotherapy being recommended by clinical practice guidelines [1, 7], or comparing the combination of YPFS and pharmacotherapy to the same pharmacotherapy. If YPFS was used together with other CHM herbs, only the studies using YPFS as the chief formula with other herbs as additional modification were included.

Studies were included if they reported one of the pre-defined outcomes, these are: the primary outcome measures being total nasal symptom score (TNSS) or individual nasal symptom scores; the secondary outcomes are effective rate (a composite outcome measure which calculates the change of nasal symptoms scores and nasal signs), QoL, recurrence rate in follow-up phase, serum specific immunoglobulin E (sIgE) level, serum interleukin 4 (IL-4), and adverse events (AEs).

### Search strategy

A comprehensive search was conducted in seven major English and Chinese databases from their inceptions to September 2017. Searched databases are: PubMed, Cochrane Central Register of Controlled Trials (CENTRAL), EMBASE, Chinese Biomedicine (CBM), China Network Knowledge Infrastructure (CNKI), Wanfang Database and Chinese Scientific Journals Database (VIP). Reference lists of all full text articles were hand-searched for additional studies. The ongoing trials were searched from clinical trial registries. The search strategies for PubMed and Chinese Databases are provided as examples (see Additional file 2). Abstracts and full texts were screened for eligibility by two authors (QL and CSZ) independently according to the selection criteria. Disagreement was solved through discussion with the third author (LY).

### Data extraction

Data were extracted and entered into a pre-defined Excel spread sheet, including study design, participants’ demographic data, details of CHM and control treatments, outcome data and AEs.

### Methodological assessment

Two authors (QL and LY) assessed the methodological quality of the included studies independently using the risk of bias tools according to the Cochrane Handbook version 5.1.0 [12]. Disagreements were resolved through discussion with another author (XG). Each item was assessed as low, high or unclear risk, with reasons recorded to support the judgments.

### Statistical analysis

The RevMan 5.3 software was used for performing meta-analyses. Studies were grouped for analyses according to their comparisons and outcome measures. Dichotomous data were expressed as risk ratio (RR) and continuous data were presented as mean difference (MD), both with 95% confidence intervals (CI). A fixed-effect model was used when heterogeneity (I^2^) was less than 50%; otherwise a random effects model was applied. Subgroup analysis was performed based on the type of pharmacotherapy medicine used in the control groups (e.g., second-generation antihistamine, intranasal glucocorticosteroids) and treatment duration (two weeks or more than three weeks). Publication bias was assessed using a funnel plot and Egger’s test where more than ten trials in a meta-analysis.

#### GRADE

The Grading of Recommendations Assessment, Development and Evaluation (GRADE) approach [13] was used to summarize and rate the quality of evidence. Summary of Findings (SOF) tables were prepared using the online software program “GRADE pro GDT” (https://gradepro.org/). The main comparisons being assessed using GRADE methods were identified through discussion.

## Results

**Fig. 1:**
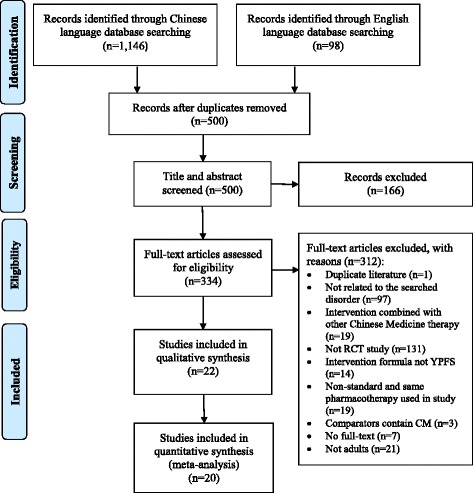
Flow chart of study selection process

In total, 1244 records were obtained through database searches, and 334 potentially relevant articles were identified after screening titles and abstracts. According to the selection criteria, 312 articles were further excluded. Twenty-two RCTs were included in the review [14–35], twenty of them were included meta-analyses [16–35]. Twenty-one included trials were conducted in the mainland China and published in Chinese [15–35], and one was conducted in China Hong Kong and published in English [14]. The search and study selection process is shown in Fig. 1, and the characteristics of included RCTs are presented in Table 1.

**Table 1:** Characteristics of included RCTs

First author, publication year, country	Treatment duration, follow-up duration	Duration of AR (mean (SD))	Number of participants of randomized/ assessed	Age (mean (SD) or range in years), gender (M/F)	Intervention (route of administration)	Control (route of administration, dosage)	Adverse events (number of events)
1 YPFS vs placebo							
Chan 2014 [14], Hong Kong China	4 weeks, 3 months	I: >1 year; C: >1 year	I: 83/80; C:83/79	I: 18–25, 32/48; C: 18–25, 35/44	Modified YPFS (oral)	Placebo (oral, 70 ml each time, once per day)	Increased acne and abdominal distension (4)
Feng 2004 [15], China	8 weeks, NS	I:10.57 (7.31) years C:11.31 (8.17) years	I: 43/43; C: 43/43	I: 37.74 (14.57), 22/21; C: 38.17 (14.21), 22/21	Allergic Rhinitis oral liquid (oral)	Saline (oral, 20 ml each time, three times a day)	NS
2 YPFS vs pharmacotherapy							
Fang 2014 [16], China	1 month, NS	I: 3.26 (1.27) years; C: 3.17 (1.12) years	I: 118/118; C: 102/102	I: 35.18 (13.72), 53/65; C: 36.03(12.85), 43/59	*Yu ping feng* capsule (oral)	Cetirizine Hydrochlorde (oral, 10 mg once per day), Budesonide aerosol (nasal spray, twice per day)	No AE
Wang 2014 [17], China	14 days, NS	I: NS; C: NS	I: 30/30; C: 30/30	I: 33 (10.78), 14/16; C:35 (11.94), 15/15	Modified YPFS (oral)	Cetirizine Hydrochloride (oral, 10 mg once per day)	NS
Luo 2012 [18], China	28 days, 1 year	I: 4.34 (4.29) years C: 4.52 (4.45) years	I: 60/60; C: 60/60	I: 34.64 (11.35), 38/22; C: 35.20 (10.43), 34/26	Modified YPFS (oral)	Cetirizine Hydrochloride (oral, 10 mg once per day)	NS
Wu 2013 [19], China	14 days, 1 year	I: 5 (1–11) years; C: 6 (1–8) years	I: 50/50; C: 50/50	I: 36, 22/28; C: 39, 27/23	*Yu ping zhi ti* granule (oral)	Loratadine (oral, 10 mg before bedtime)	NS
Wang 2009 [20], China	14 days, 1 month	I: 4.34 (4.29) years; C: 4.52 (4.45) years	I: 30/30; C: 30/30	I: 34.64 (11.35), 19/11; C: 35.2 (10.43), 17/13	Modified YPFS (oral)	Cetirizine Hydrochloride (oral, 10 mg once per day)	NS
Guo 2015 [21], China	15 days, NS	I: NS; C:NS	I: 35/35; C: 35/35	I: 26.5 (6.3), 20/15; C: 32 (4.3), 16 /19	*Yu ping feng* granule (oral)	Cetirizine Hydrochloride (oral, 10 mg once per day)	NS
Yao 2015 [22], China	4 weeks, NS	I: 8. 8(3.5) years; C: 8.6 (3.1) years	I: 43/43; C: 40/40	I: 41.8 (5.5), 18/25; C: 40.3 (4.9), 17 /23	Modified YPFS (oral)	Cetirizine Hydrochlorde (oral, 10 mg before bedtime)	No AE
3 YPFS + pharmacotherapy vs pharmacotherapy							
Shi 2014 [24], China	28 days, No follow up	I: 4.18 (2.82) years; C: 4.67 (2.33) years	I: 44/44; C: 32/32	I: 36.5 (17.5), 23/21 C: 37.5 (16.5), 17/15	*Yu ping feng* drop pill and Cetirizine (oral)	Cetirizine (oral, 10 mg once per day)	No AE
Lu 2014 [25], China	2 weeks, 8 weeks	I: (1 month-3 years); C: (1 month-2.5 years)	I: 40/40 C: 40/40	I: 40.3 (10.5), 28/12; C: 38.0 (11.2), 27/13	*Yu ping feng* granule (oral) + Ebastine (oral), Fluticasone propionate (nasal spray)	Ebastine (oral, 10 mg once per day), Fluticasone propionate (nasal spray, two sprays each nostril, once per day)	No AE
Zhou 2010 [26], China	1 month; NS	I: NS; C:NS	I: 36/36; C: 36/36	I: 45.5, 15/21; C: 43.6, 15/21	*Yu ping feng* granule (oral) + Budesonide aerosol (nasal spray)	Budesonide aerosol (nasal spray, two sprays each nostril, twice per day)	No AE
Huang 2014 [27], China	4 weeks; 3 months	I: 12.7 (3.1) months; C:11.2 (2.5) months	I: 74/74; C: 71/71	I: 30.3 (3.4), 40/34; C: 31.2 (2.7), 38/33	Modified YPFS (oral) + Cetirizine Hydrochloride (oral); Budesonide aerosol (nasal spray)	Cetirizine Hydrochloride (oral, one spray each nostril, twice per day), Budesonide aerosol (nasal spray, 10 mg once per day)	NS
Liu 2012 [28], China	30 days; NS	I: 4 years; C: 4 years	I: 30/28; C: 30/29	I: 39.25 (5.58), 13/15; C: 39.97 (4.15), 15/14	Modified YPFS + Loratadine (oral)	Loratadine (oral, 10 mg once per day)	No AE
Chen 2014 [29], China	28 days; 6 months	I: NS; C: NS	I: 60/60; C: 60/60	I: NS, NS; C: NS, NS	Modified YPFS + Cetirizine (oral)	Cetirizine (oral, 10 mg once per day)	I: skin rash (2), vomit (1), nausea (2); C:skin rash (5), vomit (4), nausea (3)
Lin 2014 [30], China	30 days; NS	I: 4.25 (1) years; C:4.28 (1.05) years	I: 30/28; C: 30/29	I:33.18 (11.42), 16/12; C:33.89 (9.35), 16/13	Modified YPFS + Cetirizine (oral)	Cetirizine (oral, 10 mg once per day)	No AE
Guan 2013 [31], China	14 days; NS	I: NS; C: NS	I: 27/27; C: 26/26	I: NS, NS; C: NS, NS	YPFS (oral) + Budesonide aerosol (nasal spray)	Budesonide aerosol (nasal spray, 64μg each nostril, twice per day)	I: sedation (1), local mucosa irritation (2); C: local mucosa irritation (2), skin rash (2)
Chen 2015 [32],China	21 days; NS	I: 5.92(4.07) years; C: 6.16(4.39) years	I: 63/63; C: 63/63	I:35.36 (7.19), 35/28; C:34.18 (6.82), 33/30	Modified YPFS (oral) + Azelastine (nasal spray);	Azelastine (nasal spray, one spray each nostril, twice per day)	NS
Ma 2017 [33],China	4 weeks; NS	I: 5.1(0.3) years; C: 5.3(0.6) years	I: 37/37; C: 37/37	I: 38.6 (6.9), 21/16; C: 37.9 (6.5), 20/17	*Yu ping feng* granule (oral) + Azelastine (nasal spray);	Azelastine (nasal spray, one spray each nostril, twice per day)	NS
Qiu 2017 [34],China	2 weeks; NS	I: 7.83(1.35) years; C: 7.68(1.40) years	I: 50/50; C: 50/50	I: 35.41 (7.35),28/22; C: 36.07 (7.18),29/21	*Yu ping feng* granule (oral) + Ebastine (oral);	Ebastine (oral, 10 mg once per day)	NS
Zheng 2017 [35],China	4 weeks; NS	I: 7.7(1.3) years; C: 8.1(1.5) years	I: 36/36; C: 36/36	I: 42.7 (12.3),18/18; C: 43.1 (11.6),20/16	*Yu ping feng* granule (oral) + Levocetirizine (oral);	Levocetirizine (oral, 5 mg once per day)	No AE
4 YPFS + pharmacotherapy vs pharmacotherapy vs YPFS							
Yu 2012 [23], China	14 days; NS	I: 2.7 (0.5) years; C1: 2.8 (0.4) years; C2: 2.5 (0.6) years	I: 60/60; C1: 60/60; C2: 60/60;	I:32.9 (6.4), 33/27; C1: 34.0 (5.9), 31/29; C2: 33.0 (6.1), 32/28	YPFS plus *Cang er zi san* (oral)	C1: Azelastine Hydrochloride (nasal spray, one spray each nostril, twice per day); C2: YPFS plus *Cang er zi san* (oral) + Azelastine Hydrochloride (nasal spray, one spray each nostril, twice per day)	I: no AE; C1 and C2: dryness in the nasal cavity (6)

Note: RCT: randomized controlled trial; YPFS: *yu ping feng san*; M/F: male/female; I: intervention group; C: control group; NS: not stated; SD: standard deviation; AE: adverse event

### Participants

In total, 2309 participants with AR were involved in the 22 RCTs, 309 participants were diagnosed with perennial allergic rhinitis, and 2000 participants were AR without specified subtype. All participants aged between 18 to 70 years. Of the reported gender information, there were a total of 1153 males and 1098 females. Participants were allocated to the YPFS group (*n* = 552), placebo group (*n* = 126), pharmacotherapy group (*n* = 1044) and the combination of CHM and pharmacotherapy group (*n* = 587). Five studies did not provide information on participants’ duration of disease [17, 21, 26, 29, 31], the remaining 17 RCTs reported the duration of disease with a median of 4.5 years.

### Interventions

Nine studies employed the original YPFS formula of only three herbs as treatment [16, 21, 24–26, 31, 33–35], the remaining 13 studies used modified YPFS formulas. The dosage and composition of YPFS formulas used in different studies are presented in Table 2. The preparation forms of the CHM were decoctions (10 studies) [17, 18, 20, 22, 23, 27–30, 32], granule (eight studies) [19, 21, 25, 26, 31, 33–35], capsule (one study) [16], pill (one study) [24], and liquid extract (two studies) [14, 15]. In regards to the comparator, two studies compared YPFS to placebo [14, 15], seven studies compared YPFS to pharmacotherapy [16–22], 12 studies compared the combination of YPFS and pharmacotherapy to pharmacotherapy alone [24–35], and one three-arm study [23] contained both pharmacotherapy and YPFS plus pharmacotherapy as controls. In addition, two studies had an arm of a different CHM formula which are not included in our review [14, 15].

**Table 2:** CHM used in all included studies

First author, publication year, country	Name	Form of the CHM	Ingredients (dosage)	Packaging dosage; Dosage and times of administration	Modification
Chan 2014 [14], Hong Kong China	Modified YPFS	Syrup	*Huangqi* 15 g, *baizhu* 12 g, *fangfeng* 3 g*, xinyi 3 g, cangerzi* (NS)*, gancao* (NS).	NS; 70 ml of syrup once a day	Add *xinyi* 3 g*, cangerzi* (NS)*, gancao* (NS).
Feng 2004 [15], China	Allergic Rhinitis oral liquid	Oral liquid	*Huangqi* (NS), *baizhu* (NS), *fangfeng* (NS)*, guizhi* (NS)*, baishao* (NS)*, dazao* (NS)*, shanyao* (NS)*, fuling* (NS)*, baibiandou* (NS)*, xinyi* (NS)*, cangerzi* (NS)*, mudanpi* (NS)*, gancao* (NS).	10 ml each bottle; 20 ml each time, three times per day	Add *guizhi* (NS)*, baishao* (NS)*, dazao* (NS)*, shanyao* (NS)*, fuling* (NS)*, baibiandou* (NS)*, xinyi* (NS)*, cangerzi* (NS)*, mudanpi* (NS)*, gancao* (NS).
Fang 2014 [16], China	*Yu ping feng* capsule	Capsule	*Huangqi* (NS), *baizhu* (NS), *fangfeng* (NS).	0.5 g each capsule, 20 capsules in one box; Two capsules each time, three times per day	None
Wang 2014 [17], China	Modified YPFS	Decoction	*Huangqi* 10 g, *baizhu* 10 g, *fangfeng* 10 g, *guizhi* 6 g*, cangerzi* 10 g*, xinyi* 10 g*, baizhi* 6 g*, wumei* 10 g*, gancao* 6 g.	NS; One dosage for twice per day	Add *guizhi* 6 g*, cangerzi* 10 g*, xinyi* 10 g*, baizhi* 6 g*, wumei* 10 g*, gancao* 6 g.
Luo 2012 [18], China	Modified YPFS	Decoction	*Huangqi* 20 g, *baizhu* 15 g, *fangfeng* 9 g*, dangshen* 20 g*, fuling* 15 g*, dilong* 12 g*, wuweizi* 12 g*, jingjie* 9 g*, xinyi* 9 g*, chantui* 9 g*, ganjiang* 6 g*, xixin* 6 g*, gancao* 6 g.	NS; One dosage each time, twice per day	Add *dangshen* 20 g*, fuling* 15 g*, dilong* 12 g*, wuweizi* 12 g*, jingjie* 9 g*, xinyi* 9 g*, chantui* 9 g*, ganjiang* 6 g*, xixin* 6 g*, gancao* 6 g.
Wu 2013 [19], China	*Yu ping zhi ti* granule	Granule	*Huangqi* 15 g, *baizhu* 10 g, *fangfeng* 10 g*, jiangcan* 10 g*, chantui 9 g, jianghuang* 6 g*, dahuang* 3 g*, xinyi* 9 g.	One herb granule in one small sized sachet; One sachet each time, twice per day	Add *jiangcan* 10 g*, chantui* 9 g*, jianghuang* 6 g*, dahuang* 3 g*, xinyi* 9 g.
Wang 2009 [20], China	Modified YPFS	Decoction	*Huangqi* 20 g, *baizhu* 15 g*,* *fangfeng* 6 g*, baijili* 10 g*, jingjie* 10 g*, xixin* 3 g.	NS; One dosage per day	Add *baijili* 10 g*, jingjie* 10 g, *xixin* 3 g.
Guo 2015 [21], China	*Yu ping feng* granule	Granule	*Huangqi* (NS), *baizhu* (NS), *fangfeng* (NS).	5 g each sachet; One sachet each time, three times per day	None
Yao 2015 [22],China	Modified YPFS	Decoction	*Huangqi* 60 g, *baizhu* 20 g, *fangfeng* 15 g, *cangerzi* 10 g, *xinyi* 10 g, *jingjie* 15 g, *baizhi* 15 g*,manjingzi* 10 g, *fuling* 20 g, *sharen* 10 g.	NS; 100 ml each time, twice per day	Add *cangerzi* 10 g, *xinyi* 10 g, *jingjie* 15 g, *baizhi* 15 g*, manjingzi* 10 g, *fuling* 20 g, *sharen* 10 g.
Yu 2012 [23], China	YPFS plus *Cang er zi san*	Decoction	*huangqi* 30 g, *baizhu* 10 g*,* *fangfeng* 10 g*, xixin* 9 g, *cangerzi* 15 g*, huangqin* 12 g*, baizhi* 20 g*, jingjie* 10 g*, shichangpu*15g*, guizhi*5g.	NS; One dosage each time, three times per day	Add *xixin* 9 g, *cangerzi* 15 g*, huangqin* 12 g*, baizhi* 20 g*, jingjie* 10 g, *shichangpu* 15 g*, guizhi* 5 g*.*
Shi 2014 [24], China	*Yu ping feng* drop pill	Drop pill	*Huangqi* (NS), *baizhu* (NS), *fangfeng* (NS).	2.4 g each sachet; One sachet each time, three times per day	None
Lu 2014 [25], China	*Yu ping feng* granule	Granule	*Huangqi* (NS), *baizhu* (NS), *fangfeng* (NS).	5 g each sachet; One sachet each time, three times per day	None
Zhou 2010 [26], China	*Yu ping feng* granule	Granule	*Huangqi* (NS), *baizhu* (NS), *fangfeng* (NS).	5 g each sachet; One sachet each time, three times per day	None
Huang 2014 [27], China	Modified YPFS	Decoction	*Huangqi* 30 g, *baizhu* 10 g, *fangfeng* 10 g*, shanyao* 15 g*, zexie* 12 g*, zhiqiao* 12 g*, cheqianzi* 12 g*, shengma* 12 g*, cangerzi* 10 g*, xinyi* 10 g*, chaihu* 10 g*, hezi* 10 g*, dilong* 8 g.	NS; One dosage for twice per day	Add *shanyao* 15 g*, zexie* 12 g*, zhiqiao* 12 g*, cheqianzi* 12 g*, shengma* 12 g, *cangerzi* 10 g*, xinyi* 10 g*, chaihu* 10 g*, hezi* 10 g*, dilong* 8 g.
Liu 2012 [28], China	Modified YPFS	Decoction	*Huangqi* 30 g, *baizhu* 12 g*,* *fangfeng* 12 g, *cangerzi* 9 g*, xinyi* 9 g*, baizhi* 9 g*, zisu* 9 g*, gancao* 6 g.	NS; One dosage each time, twice per day	Add *cangerzi* 9 g*, xinyi* 9 g*, baizhi* 9 g*, zisu* 9 g*, gancao* 6 g.
Chen 2014 [29], China	Modified YPFS	Decoction	*Huangqi* 30 g, *baizhu* 20 g, *fangfeng* 12 g*, dangshen* 20 g*, danggui* 15 g, *xinyi* 12 g*, gancao* 12 g.	NS; 200 ml of one dosage, once per day	Add *dangshen* 20 g*, danggui* 15 g, *xinyi* 12 g*, gancao*12g.
Lin 2014 [30], China	Modified YPFS	Decoction	*Huangqi* 30 g, *baizhu* 30 g*,* *fangfeng* 15 g, *baizhi 10 g, xixin 3 g.*	NS; One dosage each time, twice per day	Add *baizhi10g, xixin* 3 g*.*
Guan 2013 [31], China	YPFS	Granule	*Huangqi* (NS), *baizhu* (NS), *fangfeng* (NS).	5 g each sachet; One sachet each time, three times per day	None
Chen 2015 [32], China	Modified YPFS	Decoction	*Huangqi* 30 g, *baizhu* 15 g, *fangfeng* 15 g, *cangerzi* 9 g*,baizhi* 9 g*, gancao* 6 g*, xixin* 3 g*, zisu* 9 g.	NS; 200 ml each time, twice per day	Add *cangerzi* 9 g*, baizhi* 9 g*, gancao* 6 g*, xixin* 3 g*, zisu* 9 g.
Ma 2017 [33], China	*Yu ping feng* granule	Granule	*Huangqi* (NS), *baizhu* (NS), *fangfeng* (NS).	15 g each sachet; 5 g each time, three times per day	None
Qiu 2017 [34], China	*Yu ping feng* granule	Granule	*Huangqi* (NS), *baizhu* (NS), *fangfeng* (NS).	5 g each sachet; 5 g each time, three times per day	None
Zheng 2017 [35], China	*Yu ping feng* granule	Granule	*Huangqi* (NS), *baizhu* (NS), *fangfeng* (NS).	NS; One sachet each time, three times per day	None

Note: YPFS: *yu ping feng san*; g: gram; ml: milliliters; NS: not stated

Pharmacotherapy being used as comparators were: 1) oral [17–22, 24, 28–30, 34, 35] or intranasal [23, 32, 33] second generation H1-antihistamines, 2) intranasal glucocorticosteroids [26, 31], and 3) the combination of second generation H1-antihistamines and intranasal glucocorticosteroids [16, 25, 27].

### Outcomes

The primary outcome measures of individual nasal symptom scores were reported in four studies [29, 32, 34, 35]. In terms of the secondary outcome measures, effective rate was reported in 20 studies [15–32, 34, 35], with two methods of calculating the effective rate being used as stated in Chinese clinical guidelines [36–39]. Patients’ QoL using Rhinoconjunctivitis Quality of Life Questionnaire (RQLQ) score was assessed by one study [14]. Reported laboratory tests results were serum sIgE [15] and IL-4 [15, 16, 30, 33, 34]. In addition, the recurrence rate in follow-up phase was reported in two studies [18, 20].

### Treatment duration and follow-up

All included studies provided treatment of equal duration to the intervention and control groups. The treatment duration was two weeks or fifteen days in eight studies [17, 19–21, 23, 25, 31, 34], four weeks or one month in 12 studies [14, 16, 18, 22, 24, 26–30, 33, 35], three weeks [32] and eight weeks [15] each in one study. Six studies [14, 18, 20, 25, 27, 29] reported a post treatment follow-up phase, being one month [20], eight weeks [25], three month [14, 27], six months [29] and one year [18].

### Dropouts

Three studies reported dropouts during treatment phase with reasons provided [14, 28, 30]. None of the dropouts was due to serious AE. The other 19 studies were considered to have no dropouts since they reported equal numbers of participants at randomization and study completion.

### Risk of bias assessment

Eight studies were assessed as “low” risk for “sequence generation” since they used random number “tables” [14, 16, 20, 22, 23, 29, 33, 35], others were “unclear”. For “allocation concealment”, one study was “low” risk because it used a sealed envelope method [14], others were “unclear” due to lack of information. One study employed placebo control and made effort to blind participants, personnel and outcome assessors, thus this study was assessed as “low” risk for these items [14]; the other 21 studies were “high” risk for “blinding of participants and personnel” and “unclear” risk for “blinding of outcome assessors”. All studies were “low” risk for the “incomplete outcome data”, since they were considered having no dropouts [15–27, 29–35], or reported similar number of dropouts from both groups which was unlikely to cause bias [14, 28, 30]. One study reported all outcomes pre-defined in its registered protocol therefore it received a “low” risk for the “selective outcome reporting” [14], others were “unclear”. Due to lacking of information, all studies were “unclear” for “other bias” focusing on the funding resources.

### Effectiveness

The effectiveness of YPFS were firstly analysed according to the comparators as follow. All meta-analyses results are summarised in Table 3.

**Table 3:** Summary of meta-analyses results

Intervention vs control	Overall analysis or subgroup analysis	Subgroup details	Outcome measure	Number of studies	References	Number of participants	Results	I^2^
YPFS vs Pharmacotherapy	Overall	N/A	Effective rate	8	[16–23]	833	RR1.07, 95%CI [0.94, 1.22]	70%
YPFS vs Pharmacotherapy	Subgroup analysis (Pharmacotherapy)	Second-generation antihistamines	Effective rate	7	[17–23]	613	RR1.04, 95%CI [0.90, 1.19]	64%
YPFS vs Pharmacotherapy	Subgroup analysis (treatment duration)	Two weeks	Effective rate	5	[17, 19–21, 23]	410	RR1.04, 95%CI [0.92, 1.18]	6%
YPFS vs Pharmacotherapy	Subgroup analysis (treatment duration)	Three weeks or more	Effective rate	3	[16, 18, 22]	423	RR1.08, 95%CI [0.82, 1.42]	90%
YPFS plus pharmacotherapy versus pharmacotherapy	Overall	Second-generation antihistamines	Itchy nose	4	[29, 32, 34, 35]	418	MD-0.46, 95%CI [−0.50, −0.42]*	0
YPFS plus pharmacotherapy versus pharmacotherapy	Overall	Second-generation antihistamines	Sneezing	4	[29, 32, 34, 35]	418	MD-0.41, 95%CI [−0.47, −0.35]*	54%
YPFS plus pharmacotherapy versus pharmacotherapy	Overall	Second-generation antihistamines	Blocked nose	4	[29, 32, 34, 35]	418	MD-0.46, 95%CI [−0.54, −0.39]*	60%
YPFS plus pharmacotherapy versus pharmacotherapy	Overall	Second-generation antihistamines	Runny nose	3	[29, 32, 35]	318	MD-0.42, 95%CI [−0.58, −0.26]*	70%
YPFS plus pharmacotherapy versus pharmacotherapy	Overall	N/A	Effective rate	12	[23–32, 34, 35]	1077	RR1.27, 95%CI [1.19, 1.34]*	22%
YPFS plus pharmacotherapy versus pharmacotherapy	Subgroup analysis (Pharmacotherapy)	Second-generation antihistamines	Effective rate	8	[23, 24, 28–30, 32, 34, 35]	727	RR1.28, 95%CI [1.19, 1.37]*	43%
YPFS plus pharmacotherapy versus pharmacotherapy	Subgroup analysis (Pharmacotherapy)	Second-generation antihistamine + intranasal glucocorticosteroids	Effective rate	2	[25, 27]	225	RR1.29, 95%CI [1.14,1.46]*	0
YPFS plus pharmacotherapy versus pharmacotherapy	Subgroup analysis (Pharmacotherapy)	Intranasal glucocorticosteroids	Effective rate	2	[26, 31]	125	RR1.15, 95%CI [0.97, 1.36]	0
YPFS plus pharmacotherapy versus pharmacotherapy	Subgroup analysis (treatment duration)	Two weeks	Effective rate	4	[23, 25, 31, 34]	353	RR1.13, 95%CI [0.84, 1.54]	88%
YPFS plus pharmacotherapy versus pharmacotherapy	Subgroup analysis (treatment duration)	Three weeks or more	Effective rate	8	[24, 26–30, 32, 35]	725	RR1.15, 95%CI [1.01,1.32]*	74%

Note: YPFS: *yu ping feng san*; RR: risk ratio; MD: mean difference; CI: Confidence interval. *: significant difference between two groups was detected

#### YPFS versus placebo

Two studies compared YPFS with oral placebo [14, 15]. Meta-analysis was not possible because they reported data on different outcomes.

One study reported data on RQLQ, it was found that the post-treatment total RQLQ score of the YPFS group was lower than that of the placebo group (*n* = 159, MD-8.57, 95%CI [−16.37, −0.77]) [14]. Another study reported post-treatment data on effective rate, serum sIgE and IL-4. Significant difference between the YPFS group and the placebo group was found for all these outcomes (*n* = 86, effective rate: RR13.33, 95%CI [4.46, 39.83]; sIgE: MD-16.18, 95%CI [−22.50, −9.86]; IL-4: MD-19.65, 95%CI [−25.32,-13.98]) [15]. However, these results are only from single study, the small sample size and wide confidence interval makes the results unconvinced.

#### YPFS versus pharmacotherapy

Seven studies compared YPFS to pharmacotherapy [16–22], and one three-arm study also contained this comparison [23]. None of these studies reported nasal symptom scores.

**Fig. 2:**
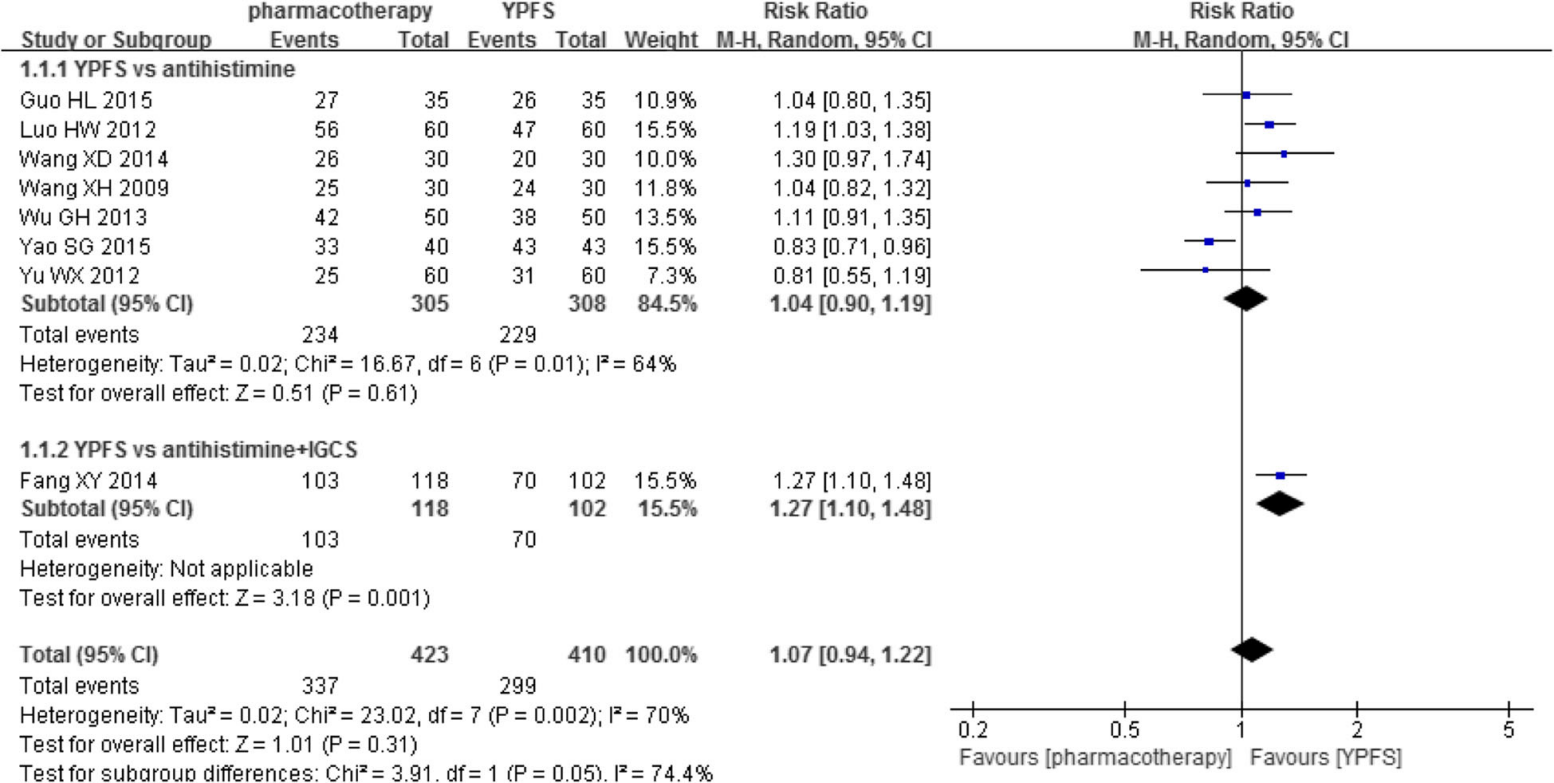
Forest plot: YPFS versus pharmacotherapy (effective rate based on pharmacotherapy). Abbreviation: IGCS: intranasal glucocorticosteroids

All these studies reported post-treatment data on effective rate, the overall meta-analysis showed that YPFS did not achieve superior effect than overall pharmacotherapy (*n* = 833, RR1.07, 95%CI [0.94, 1.22], I^2^ = 70%) [16–23]. Subgroup analyses were conducted to further explore the effectiveness of YPFS comparing to different types of pharmacotherapy. It was found that, when comparing YPFS to the second-generation antihistamines, there was no significant difference between two groups for the outcome of effective rate (*n* = 613, RR1.04, 95%CI [0.90, 1.19], I^2^ = 64%) [17–23]. On the other hand, one study showed that YPFS was more effective than the combination of the second-generation antihistamine and intranasal glucocorticosteroids (*n* = 220, RR1.27, 95%CI [1.10, 1.48]) [16] (Fig. 2).

**Fig. 3:**
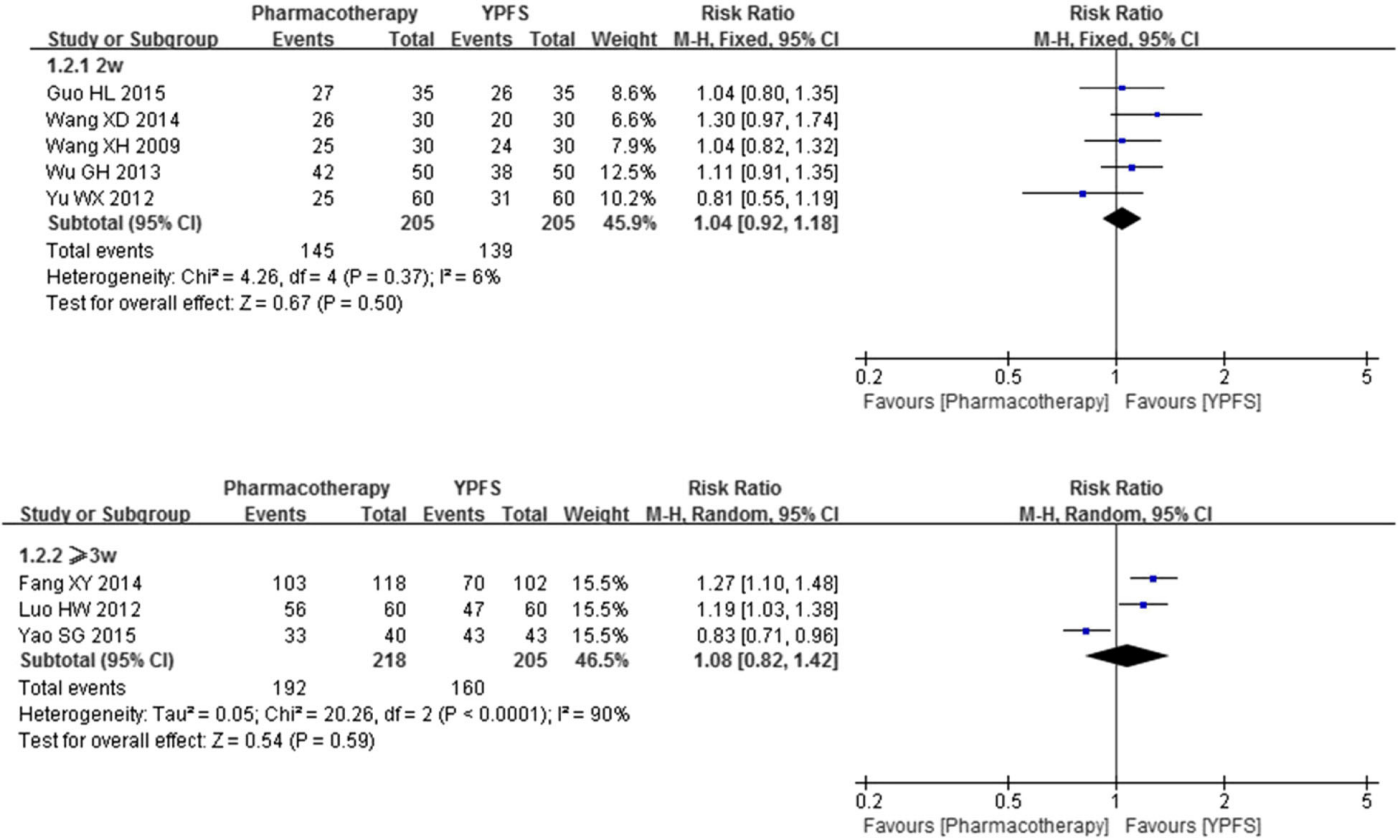
Forest plot: YPFS versus pharmacotherapy (effective rate based on treatment duration). Legends: 2w: two weeks; ≥3w: three weeks or more than three weeks

Subgroup analysis based on treatment duration was also conducted. Results showed that, in terms of the effective rate, there was no significant difference between the YPFS and pharmacotherapy when it was tested for two weeks (*n* = 410, RR1.04, 95%CI [0.92, 1.18], I^2^ = 6%) [17, 19–21, 23]; or being tested for more than three weeks (*n* = 423, RR1.08, 95%CI [0.82, 1.42], I^2^ = 90%) [16, 18, 22] (Fig. 3).

In addition, one study found that YPFS achieved better effect than intranasal glucocorticosteroids for IL-4 (n = 220, MD-37.94, 95%CI [−49.47, −26.41]) [16]. Another study reported the recurrence rate at the end of follow-up phase, it was found that the number of recurrence of the YPFS group was less than that of pharmacotherapy (Cetirizine) (*n* = 103, RR0.37, 95%CI [0.18, 0.78]) [18].

#### YPFS plus pharmacotherapy versus pharmacotherapy

Twelve studies compared the combination of YPFS and pharmacotherapy to pharmacotherapy alone [24–35], and one three-arm study also contained this comparison [23].

For the primary outcome measure, four studies using second-generation antihistamines as control reported data on individual symptoms scores [29, 32, 34, 35]. It was found that the combined intervention was more effective than second-generation antihistamines alone for four nasal symptoms: itchy nose (*n* = 418, MD-0.46, 95%CI [−0.50, −0.42], I^2^ = 0%), sneezing (n = 418, MD-0.41, 95%CI [−0.47, −0.35], I^2^ = 54%), blocked nose (n = 418, MD-0.46, 95%CI [−0.54, −0.39], I^2^ = 60%) [29, 32, 34, 35] and runny nose (*n* = 318, MD-0.42, 95%CI [−0.58, −0.26], I^2^ = 70%) [29, 32, 35].

**Fig. 4:**
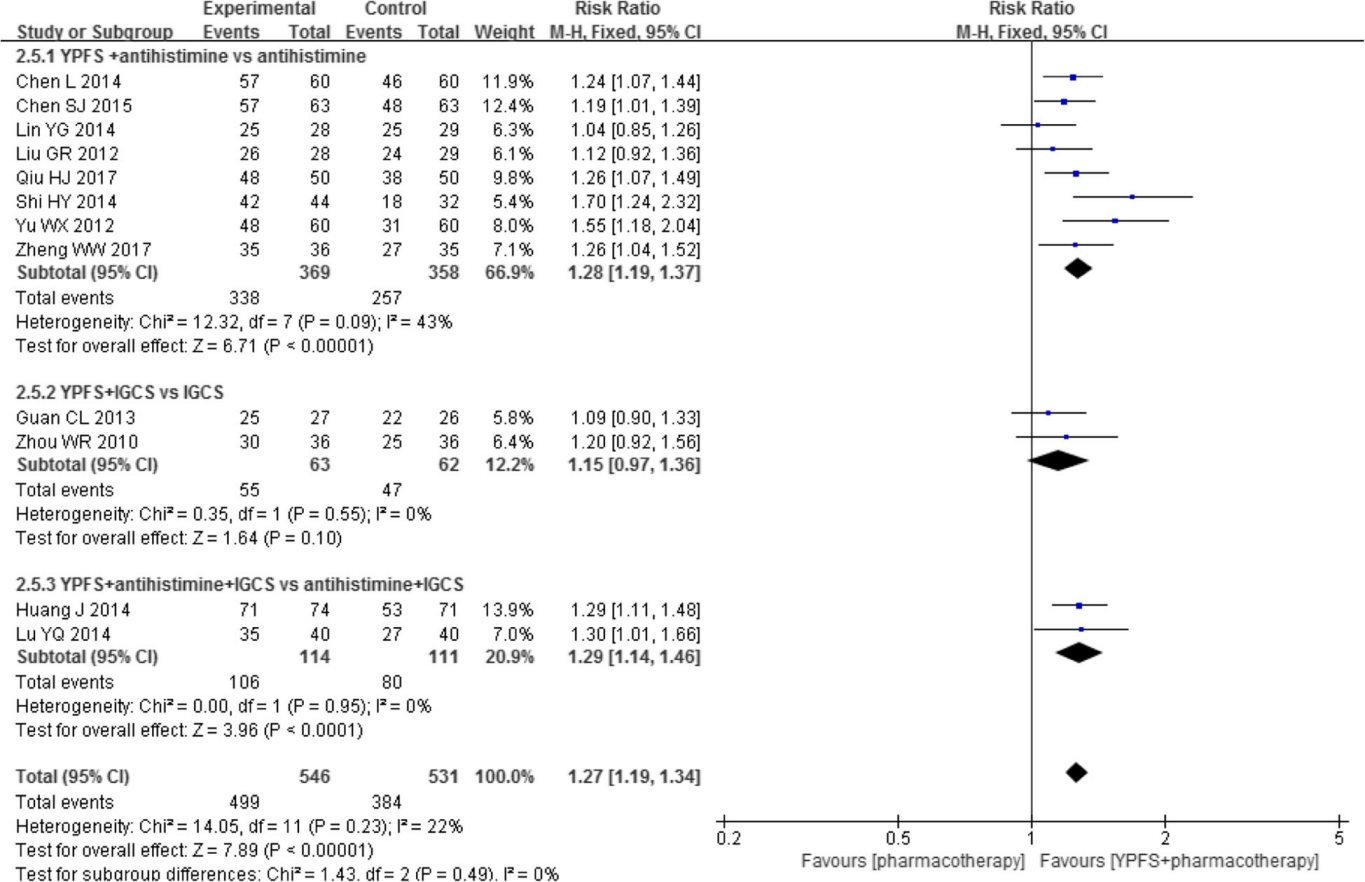
Forest plot: YPFS plus pharmacotherapy versus pharmacotherapy (effective rate based on pharmacotherapy). Abbreviation: IGCS: intranasal glucocorticosteroids.

Meta-analysis was also feasible for the outcome of effective rate. Of these 1 comparisons, meta-analysis showed that the combination was more effective than pharmacotherapy alone (*n* = 1077, RR1.27, 95%CI [1.19, 1.34], I^2^ = 22%) [23–32, 34, 35]. Subgroup analysis on effective rate was conducted according to the pharmacotherapy used in the control groups. The results showed that the combination was more effective than different types pharmacotherapy as follow: the combination versus second-generation antihistamines (*n* = 727, RR1.28, 95%CI [1.19, 1.37], I^2^ = 43%) [23, 24, 28–30, 32, 34, 35] and the combination versus the second-generation antihistamine plus intranasal glucocorticosteroids (*n* = 225, RR1.29, 95%CI [1.14,1.46], I^2^ = 0%) [25, 27]. However, the combined intervention was not superior to intranasal glucocorticosteroids alone (*n* = 125, RR1.15, 95%CI [0.97, 1.36], I^2^ = 0%) [26, 31]. (Fig. 4).

**Fig. 5:**
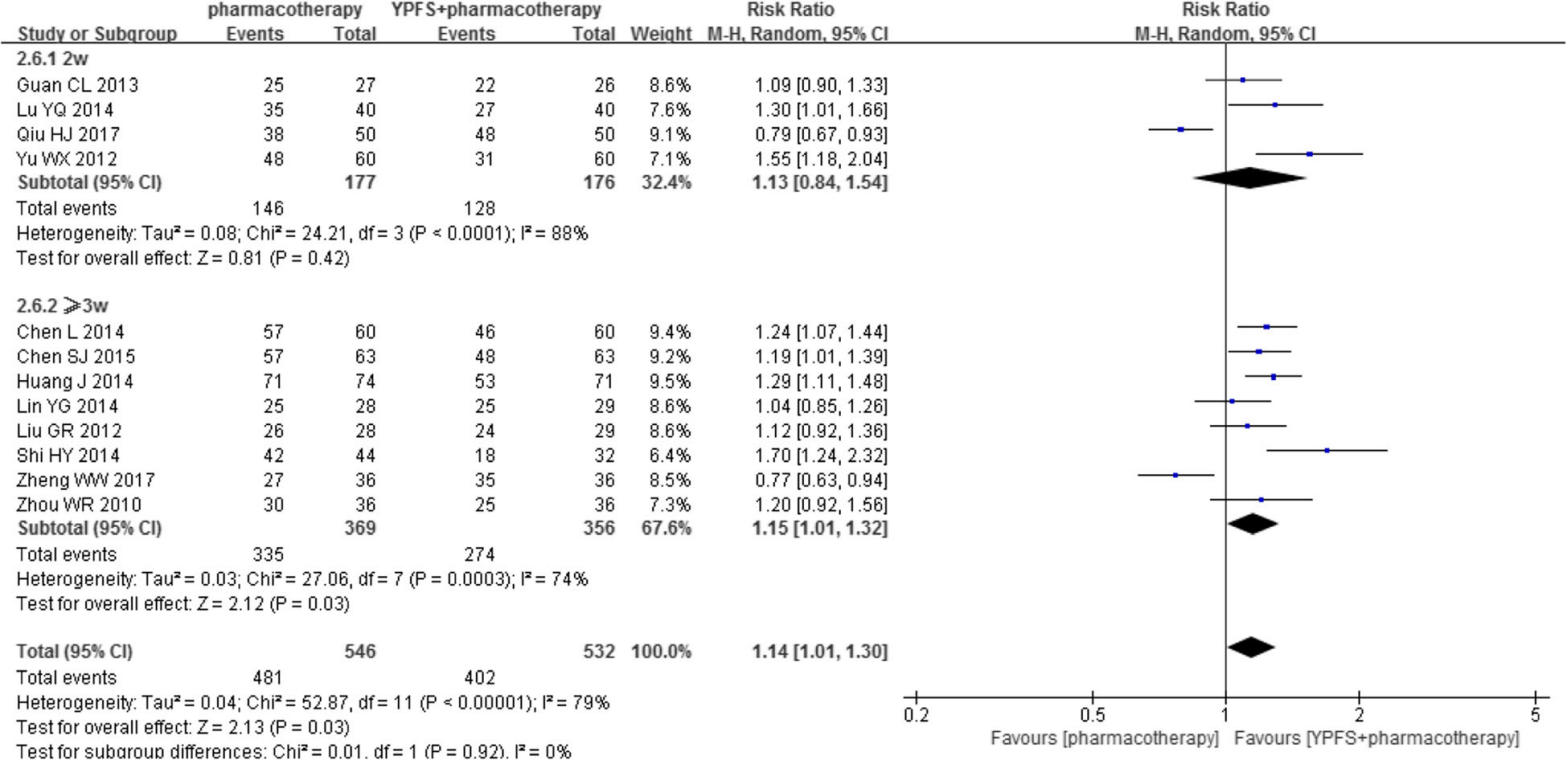
Forest plot: YPFS plus pharmacotherapy versus pharmacotherapy (effective rate based on treatment duration). Legends: 2w: two weeks; ≥3w: three weeks or more than three weeks

Subgroup analysis for effective rate was performed based on the treatment duration [23–31, 34, 35]. Superior effects towards the combination therapy was found when it was tested for more than three weeks, (*n* = 725, RR1.15, 95%CI [1.01, 1.32], I^2^ = 74%) [24, 26–30, 32, 35], but it was not found in the subgroup of two weeks treatment (*n* = 353, RR1.13, 95%CI [0.84, 1.54], I^2^ = 88%) [23, 25, 31, 34] (Fig. 5).

In addition, biomarker IL-4 was reported by three studies and it showed that the YPFS and second-generation antihistamines combination treatment were superior to second-generation antihistamines alone (*n* = 231, MD-14.43, 95%CI [−28.58, −0.29]) [30, 33, 34].

### Safety

Ten studies did not report information on AEs [15, 17–21, 27, 32–34]. Eight studies reported there was no AE occurred in both groups [16, 22, 24–26, 28, 30, 35]. The remaining four studies reported AEs in treatment group [14, 23, 29, 31], and control groups [23, 29, 31] during the treatment phase. According to these studies, the AEs possibly associated with the use of YPFS were acne and abdominal distension [14]; while the AEs possibly caused by pharmacotherapy included dryness in the nasal cavity [23], local mucosa irritation [31], sedation [31], skin rash [29, 31], vomit and nausea [29]. All above AEs were transient and no medical intervention was required. Serious AE was not reported by any study. It was worth noting that the number of AEs occurred in the combination group was less than that of the pharmacotherapy group. For example, one study reported five cases of AEs in the combination group and 12 cases in the pharmacotherapy group [29], and another study reported three cases of AEs in the combination group and four cases in the pharmacotherapy group [31]. These studies concluded that adding YPFS to pharmacotherapy is safe and it may be beneficial for reducing the AEs caused by pharmacotherapy. All detailed information of AEs is provided in Table 1.

### Publication bias

**Fig. 6:**
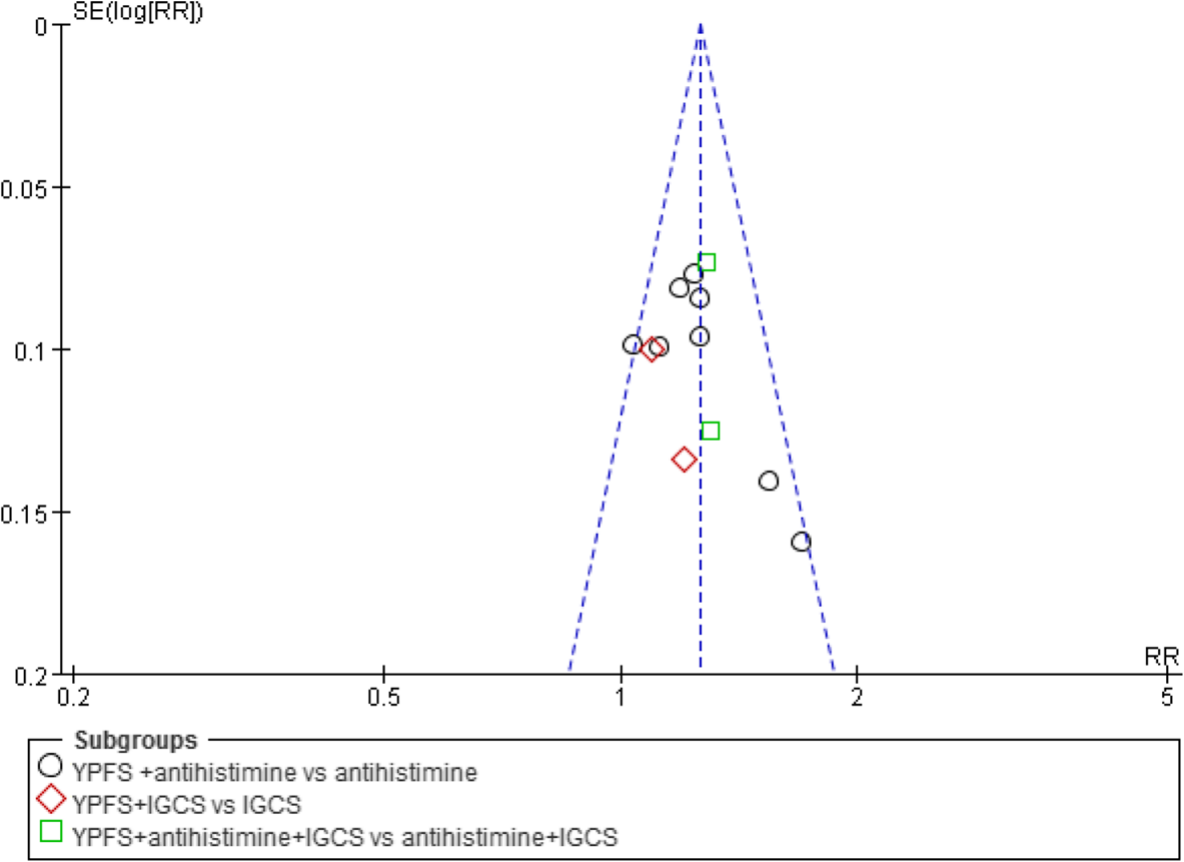
Funnel plot: YPFS plus pharmacotherapy versus pharmacotherapy (effective rate)Funnel plot: YPFS plus pharmacotherapy versus pharmacotherapy (effective rate). Abbreviation: IGCS: intranasal glucocorticosteroids

The publication bias could not be assessed for the comparison of YPFS versus pharmacotherapy since there were not enough studies included in one meta-analysis. For the comparison of YPFS plus pharmacotherapy vs. pharmacotherapy alone, there were 12 trials in the meta-analysis of effective rate. No publication bias was detected based on the funnel plot (Fig. 6) and the Egger’s test (*t* = 1.30, 95% CI [−1.26, 4.79], *p* = 0.223).

### GRADE for the main comparisons of YPFS

GRADE assessment was conducted for two main comparisons. The quality of evidence was low. Table 4 summarises the results.

**Table 4:** Summary of GRADE

Quality assessment							Number of patients		Effect		Quality	Importance
Number of studies	Study design	Risk of bias	Inconsistency	Indirectness	Imprecision	Other conside-rations	YPFS or its combi-nation	Pharmaco-therapy	Relative (95% CI)	Absolute (95% CI)		
Effective rate: YPFS vs Antihistamine for Adult Allergic Rhinitis												
7 [17–23]	Randomiz-ed trials	serious^a^	serious^b^	not serious	not serious	none	229/308(74.4%)	234/305 (76.7%)	RR 1.04 (0.90 to 1.19)	29 more per 1000 (from75 fewer to 141 more)	⨁⨁◯◯ LOW	NOT IMPORTANT
Score of Itchy nose: YPFS + Antihistamine vs Antihistamine for Adult Allergic Rhinitis												
4 [29, 32,34,35]	Randomiz-ed trials	serious^a^	not serious	not serious	serious^c^	none	209	209	MD −0.46(−0.50 to −0.42)	0.46 fewer (0.5 fewer to 0.42 fewer)	⨁⨁◯◯ LOW	IMPORTANT
Score of sneezing: YPFS + Antihistamine vs Antihistamine for Adult Allergic Rhinitis												
4 [29,32,34, 35]	Randomiz-ed trials	serious^a^	serious^b^	not serious	not serious	none	209	209	MD −0.41 (−0.47 to −0.35)	0.41 fewer (0.47 fewer to 0.35 fewer)	⨁⨁◯◯ LOW	IMPORTANT
Score of blocked nose: YPFS +Antihistamine vs Antihistamine for Adult Allergic Rhinitis												
4 [29, 32,34,35]	Randomiz-ed trials	serious^a^	serious^b^	not serious	not serious	none	209	209	MD −0.46(−0.54 to −0.39)	0.46 fewer (0.54 fewer to 0.39 fewer)	⨁⨁◯◯ LOW	IMPORTANT
Score of runny nose: YPFS +Antihistamine vs Antihistamine for Adult Allergic Rhinitis												
3 [29, 32, 35]	Randomiz-ed trials	serious^a^	serious^b^	not serious	not serious	none	209	209	MD −0.42(−0.58 to −0.26)	0.42 fewer (0.58 fewer to 0.26 fewer)	⨁⨁◯◯ LOW	IMPORTANT
Effective rate: YPFS +Antihistamine vs Antihistamine for Adult Allergic Rhinitis												
8 [23,24, 28–30,32, 34,35]	Randomiz-ed trials	serious^a^	not serious	not serious	not serious	none	338/369 (91.6%)	257/358 (71.8%)	RR 1.28 (1.19 to 1.37)	201 more per 1000 (from 136 more to 265 more)	⨁⨁◯◯ LOW	NOT IMPORTANT

*Abbreviations*: *YPFS Yu ping feng san*, *CI* confidence interval, *RR* risk ratio, *MD* mean difference

Note: ^a^Lacking of blinding, randomisation and allocation concealment are unclear. ^b^Substantial heterogeneity. ^c^Small sample size

## Discussion

This systematic review evaluated RCTs of CHM YPFS including the most recently published articles. Studies compared CHM YPFS to placebo or pharmacotherapy, and those used YPFS as add-on therapy were all included in our evaluation. The evidence of YPFS effectiveness has been revealed through meta-analyses.

### Effectiveness and safety of YPFS

In this review, meta-analyses were feasible for the outcomes of four individual nasal symptom scores and “effective rate”. Based on these outcomes, it was found that: 1) in terms of the nasal symptoms, adding YPFS to the second-generation antihistamines achieved better treatment effects (low quality evidence); 2) for “effective rate”, although YPFS was not superior to pharmacotherapy, adding YPFS to common pharmacotherapy was also more effective than using pharmacotherapy alone. Further, subgroup analysis showed that there was no significant difference between YPFS and the second-generation antihistamine in terms of “effective rate” (low quality evidence).

In order to provide evidence for the duration of clinical administration, subgroup analysis regarding the treatment duration was conducted. Our results showed that adding YPFS to pharmacotherapy was beneficial when it was administered for more than three weeks, but this was not seen in the studies of only two weeks treatment. It is worth noting that, there has been experimental research evidence showing that six days’ high and middle dosages of YPFS powder significantly increased the IFN-γ, the antibody of infectious laryngotracheitis and the immune organ indexes, and also reduced IL-4 in chickens [40]. One study reported that 14 days of oral YPFS could stimulate immune responses in allergic rhinitis mice [41]. Another study reported that after five to seven days intervention, different concentration of the glucosidic which extracted from YPFS produced anti-inflammatory and immune-regulatory effects [42]. These experimental studies indicated that YPFS alone may show immune-modulatory and anti-inflammatory effects after one to two weeks. In clinical management of AR, pharmacotherapies usually are fast-acting for symptom control but leaving 10% to 20% AR patients to endure bothersome symptoms [43, 44]. Our results suggest that, although YPFS is not superior to pharmacotherapy, adding CHM YPFS to common pharmacotherapy for at least three weeks is helpful to improve the overall symptoms. Since YPFS has been recommended as AR management by current Chinese Medicine clinical practice guideline [9], but without recommendation on the treatment duration, our results may potentially have filled this gap and provide indication for further clinical practice. Nevertheless, these finding was only based on the outcome of “effective rate” since there were not enough data on nasal symptoms scores supporting these analyses. Further studies using globally well-accepted outcome measures to assess the treatment effects at different time points are needed to confirm such findings.

Reported AEs caused by YPFS were mild and transient. It was also reported that adding YPFS could reduce the number of AEs caused by cetirizine [29] or budesonide aerosol [31], though detailed explanation was not provided by these studies. Similar findings were reported by other clinical trials of YPFS for AR management [45, 46] but without further exploration of the mechanisms. It is worth noting that some toxicity tests of YPFS also suggested that its toxicity is either minor or without any adverse effects [47, 48].

### Potential mechanisms of YPFS

Results from experimental studies showed that YPFS reduced the eosinophilic cells’ infiltration and degranulation reaction, decreased the tissue edema [49, 50], and reduced the immune factors including histamine, IgE, IL-4 and tumor necrosis factor- α (TNF-α) in serum [50]. Other researches indicated that YPFS was effective for AR through down-regulating the activity of IL-6 and TNF-α [51], activating interleukin 3 (IL-3), growth factor of mast cells, and inhibiting granulocyte colony stimulating factor (GM-SCF) and an inhibitory factor of mast cells proliferation [52]. IL-4 is one of the important Th2 cytokines related to AR, it can accelerate the synthesis of B cell and secrete a large number of IgE [7]. The result from five included studies in this review illustrated that the IL-4 level decreased after YPFS or its combination treatments [15, 16, 30, 33, 34], and one of these studies also reported the sIgE level declined after YPFS treatment [15]. Furthermore, a review article [53] suggested that YPFS may lead to immunoregulatory effects through the following pathways: 1), to promote the generation of IgA and IgG, the peripheral blood levels of CD3^+^, CD4^+^ and CD4^+^/CD8^+^; 2), to increase the amount of C3, C4 and the receptor of C3b, and activate the complement system involved in immune response; 3), to protect the phagocytosis of macrophage; and 4) to regulate the cyclic adenosine monophosphate (cAMP) in the cells of mice spleen bi-directionally.

There has been evidence showing that glucosides were the principal active ingredients of YPFS which are related to the anti-inflammatory and immunoregulatory effects [42], and claycosin and formononetin may be the effective compounds which could produce the anti-inflammatory function by reducing thymic stromal lymphopoietin production via regulating Nuclear factor (NF)-κB activation [54].

Furthermore, the three chief herbs of YPFS (*huang qi*, *fang feng*, *bai zhu*) have also been researched previously. Studies found that these herbs may have certain anti-allergy and anti-inflammatory effects [55–58]. For instance, in mice with allergen-induced airway hypersensitivity, *huang qi* injection suppressed the allergic reaction, enhanced interferon-gamma levels (important immune responsive cytokine) and decreased allergen-induced elevations of important allergy related interleukins IL-5 and IL-13 [53]. Aqueous extracts of *huang qi* inhibited nitric oxide production in lipopolysaccharide -stimulated murine macrophage RAW 264.7 cells in a dose-dependent manner and this may be associated with the inhibition of inducible nitric oxide synthase mRNA expression [56]. In RAW 264.7 cells, *fang feng* compound anomalin reduced several pro-inflammatory cytokines, including tumor necrosis factor-α and IL-6, by inhibiting NF-κB DNA binding [57]. Five *bai zhu* compounds (atractylenolide III 1, atractylenolide I 2, 14-acetoxy-12-senecioyloxytetradeca-2E,8E,10E–trien-4,6-diyn-1-ol 3, 14-acetoxy-12-alpha-methylbutyl-2E,8E,10E–trien-4,6-diyn-1-ol 4 and 14-acetoxy-12-beta-methylbutyl-2E,8E,10E–trien-4,6-diyn-1-ol 5) significantly inhibited mice ear edema induced by xylene, showing anti-inflammatory activities [58].

In brief, there has been certain pre-clinical evidence supporting the use of YPFS for AR management due to its anti-inflammatory and immunoregulatory function. More details about its mechanism and the active compounds of YPFS are yet to be explored.

### Limitations of the included studies

Proper randomization and allocation is essential to reduce selection bias for randomized controlled trials. However, in our systematic reviews, only 36.4% of the included studies (eight trials) applied appropriate methods for sequence generation, and only 4.5% (one trial) for allocation concealment. Blinding of participants, personnel and outcome measures was only achieved by one study, while other studies did not utilize proper placebo control to unsure blinding, and therefore they were not free of performance bias or detection bias.

In regards to the outcome measures, it was found the individual nasal symptom scores was reported by four studies but without TNSS being reported, and QoL was only reported by one study, while the “effective rate” was reported by the majority of the included studies. The “effective rate” assesses the proportion of participants who had achieved certain improvement of the nasal symptoms and signs. Considering that this outcome measure only reflects the overall improvement, it makes it indirect to compare the effectiveness results of these studies to other RCTs which reported data on nasal symptom scores and QoL, even though this outcome measure was recommended by several previous clinical guidelines [36–39, 59] and had been widely used in China.

Considering AR is a chronic and recurrent condition, the long-term effects or the recurrence rate after treatment is of clinicians’ interests. Two included studies reported data on the recurrence rate in follow-up phase [18, 20]. However, there is no clear definition of “recurrence” stated by these studies. Further studies should consider using the internationally well-accepted outcome measures to evaluate the long-term treatment effects of YPFS, for example, the nasal symptom scores and QoL.

In terms of clinical trial reporting, none of the included study reported all items recommended by CONSORT 2010 and its Extension for Herbal Intervention [60, 61]. Also, in the absent of trial registration information or published protocol of the included studies, evaluation of selective reporting bias was not applicable in all of the included studies.

All these limitations reduced the certainty of our results. Future research should take all these aspects into consideration and improve the methodological quality of clinical trials’ design and conduct.

### Strengths and limitations of this review

The review synthesised the most recent clinical research following rigorous methodology recommended by the Cochrane Handbook, with GRADE approach being used to assess the quality of evidence. This review focused on YPFS treating adult AR, it not only included studies comparing YPFS to placebo or pharmacotherapy, but also evaluated the add-on effects of YPFS to pharmacotherapy. Outcome measures included symptoms scores, QoL, effective rate, recurrence rate, as well as objective outcomes such as serum sIgE and IL-4.

However, a few limitations downgraded the certainty of our results, they are: 1). The majority of included studies (21 out of 22) were all conducted in China and published in Chinese. 2).outcome measures in most included studies were not consistent with international guidelines. This leads to indirectness when interpreting the findings; 3). the overall methodological quality of included studies was low; 4). the long-term effects of YPFS were uncertain due to the lack of follow-up phase of most studies.

### Implication for clinical practice and further research

The results of this review suggest that YPFS is effective for the management of adult AR as on add-on therapy. Clinical practice could consider adding oral YPFS to routine pharmacotherapy management and it may improve the effectiveness and safety of routine pharmacotherapy. A correlation of treatment duration and effects of YPFS was observed in this review, however, it requires further exploration. The mechanism of YPFS herbs and their active compounds are also worth more investigation.

Nevertheless, although YPFS in general is effective regarding individual nasal symptoms and the overall “effective rate”, but there is insufficient data supporting the effectiveness towards TNSS and QoL. Further research should consider employing outcome measures recommended by international guidelines (nasal symptom scores and QoL) to evaluate the effectiveness of YPFS. A follow-up phase with well-accepted outcome measures is also important to confirm the long-term effects of YPFS. Lack of blinding is the major methodological flaw of most included studies in this review, further research should consider adapting placebo control and making efforts to the blinding of participants and outcome assessors.

In addition, although it was seen that adding YPFS to pharmacotherapy may reduce the common AEs caused by pharmacotherapy, the mechanism of such herb-drug interaction is unknown. This may be worth exploring for future research to provide evidence of the safety aspect.

## Conclusion

Oral CHM YPFS seems to have add-on effects to pharmacotherapy for the treatment of adult AR, when it was administered for at least three weeks. Further, the use of YPFS in clinical practice is safe. Also, there has been available pre-clinical evidence supporting the use of YPFS and the three chief herbs for AR. However, the overall methodological quality of included studies was low. More RCTs following rigorous methodology and using internationally well-accepted outcome measures are needed to further define the effectiveness of YPFS.

## Additional files

Additional file 1: PRISMA 2009 checklist. (DOC 66 kb)

Additional file 2: Search Strategies. (DOCX 18 kb)

## Abbreviations

AE: Adverse event
AR: Allergic rhinitis
CHM: Chinese herbal medicine
CI: Confidence intervals
G-CSF: Granulocyte colony stimulating factor
GRADE: Grading of Recommendations Assessment, Development and Evaluation
IL-3: Interleukin 3
IL-4: Interleukin 4
MD: Mean difference
QoL: Quality of life
RCT: Randomized controlled trial
RQLQ: Rhinoconjunctivitis Quality of Life Questionnaire
RR: Risk ratio
sIgE: Specific immunoglobulin E
SOF: Summary of Findings
TNF-α: Tumor necrosis factor- α
TNSS: Total nasal symptom score
YPFS: *Yu ping feng san*

## References

[1] Bousquet J, Khaltaev N, Cruz AA, Denburg J, Fokkens WJ, Togias A (2008). Allergic rhinitis and its impact on asthma (ARIA) 2008 update (in collaboration with the World Health Organization, GA (2)LEN and AllerGen). Allergy.

[2] Dykewicz MS, Fineman S, Skoner DP, Nicklas R, Lee R, Blessing-Moore J, Li JT, Bernstein IL, Berger W, Spector S, Schuller D (1998). Diagnosis and management of rhinitis: complete guidelines of the joint task force on practice parameters in allergy, asthma and immunology. American Academy of allergy, asthma, and immunology. Ann Allergy Asthma Immunol.

[3] Skoner DP (2001). Allergic rhinitis: definition, epidemiology, pathophysiology, detection, and diagnosis. J Allergy Clin Immunol.

[4] Brozek JL, Bousquet J, Baena-Cagnani CE, Bonini S, Canonica GW, Casale TB, van Wijk RG, Ohta K, Zuberbier T, Schünemann HJ (2010). Global allergy and asthma European network and grading of recommendations assessment. Development and evaluation working group. Allergic rhinitis and its impact on asthma (ARlA) guidelines: 2010 revision. J Allergy Clin Immunol.

[5] Seidman MD, Gurgel RK, Lin SY, Schwartz SR, Baroody FM, Bonner JR (2015). Clinical practice guideline: Allergic rhinitis. Otolaryngol Head Neck Surg.

[6] Scadding GK, Durham SR, Mirakian R, Jones NS, Leech SC, Farooque S (2008). BSACI guidelines for the management of allergic and non-allergic rhinitis. Clin Exp Allergy.

[7] Bousquet J, van Cauwenberge P, Khaltaev N, Canonica GW, Khaltaev N, Carlsen KH (2001). Allergic rhinitis and its impact on asthma. J Allergy Clin Immunol.

[8] Wang SJ, Tang QF, Qian W, Fan Y (2012). Meta-analysis of clinical trials on traditional Chinese herbal medicine for treatment of persistent allergic rhinitis. Allergy.

[9] .Bi Qiu (allergic rhinitis). In: China Association of Chinese Medicine. Diagnosis and Treatment Guidelines in Traditional Chinese Medicine for Common Otolaryngology Disease. Beijing, China: Chinese Traditional Chinese Medicine Publishing House, 2012.p. 17–8. [Article in Chinese].

[10] *Yu ping feng san*. In: Li F. Prescriptions of Traditional Chinese Medicine. Beijing, China: People’s Medical Publishing House; 2002. p. 817–26. [Article in Chinese].

[11] Zhang JQ. Systematic Review of Jiawei Yupingfeng Powder on Intervention of Allergic Rhinitis Effects and Safety. J Basic Chin Med. 2016;22: 99–101. [Article in Chinese].

[12] Higgins JP, Green S. Cochrane Handbook for Systematic Reviews of Interventions Version 5.1.0. The Cochrane Collaboration, http://handbook.cochrane.org/.Acceszed 20 Mar 2011.

[13] Schunemann H, Brożek J, Guyatt G, Oxman A. (editors). GRADE handbook for grading quality of evidence and strength of recommendations (The GRADE Working Group). http://www.guidelinedevelopment.org/handbook/. Acceszed Oct 2013.

[14] Chan RY, Chien WT. The effects of two Chinese herbal medicinal formulae vs. placebo controls for treatment of allergic rhinitis: a randomized controlled trial. Trials. 2014; 10.1186/1745-6215-15-261. 10.1186/1745-6215-15-261 PMC4094638 24986270

[15] Feng WY, Wang XP, Zhang M, Wei ZZ, Fang YF. Clinical effect observation of Allergic Rhinitis Oral Liquid in treating perennial allergic rhinitis. J Guangzhou Univ Tradit Chin Med. 2004;21:101–4. [Article in Chinese].

[16] Fang XY, Feng SS, Li Y, Zhang XT, Zhu Y, Zhan G. Clinical efficacy of *Yupingfeng* capsule in treating allergic rhinitis and its safety evaluation. Chin Arch Tradit Chin Med. 2014;32: 2556–8. [Article in Chinese].

[17] Wang XD. Clinical effect observation of modified *Yu ping feng san* in treating allergic rhinitis with Lung and Spleen *qi* deficiency. Med Aesthet Beauty (midmonth). 2014;23:258. [Article in Chinese].

[18] Luo HW. Modified *Yu ping feng san* treat allergic rhinitis with Lung and Spleen deficiency-cold (60 cases). Tradit Chin Med Res. 2012;25:35–6. [Article in Chinese].

[19] Wu GH, Li L, Cao CM, Nan NY. Clinical effect observation of *Yu ping zhi ti* granule in treating allergic rhinitis. Chin J Otorhinolaryngol Integr Med. 2013;21:51–2. [Article in Chinese].

[20] Wang XH (2009). Clinical effect observation of modified *Yu ping feng san* in treating perennial allergic rhinitis.

[21] Guo HL, Liu JJ, Zhao YL. An effective analysis of treating allergic rhinitis with the *Yu ping feng* granules (35Cases). Clin J Chin Med. 2015;7(35):82–3. [Article in Chinese].

[22] Yao SG. Curative effect observation of modified *Cang er yu ping* decoction for allergic rhinitis. Chin J of Tradit Med Sci Techno. 2015;22(3):309–10. [Article in Chinese].

[23] Yu WX, Jiang YL. Clinical effect observation of Chinese and western medicine combined treatment for the treatment of allergic rhinitis. J Sichuan Tradit Chin Med. 2012;30:88–9. [Article in Chinese].

[24] Shi HY, Zhuang Y, Wang XY. Clinical effect observation of *Yu ping feng* droppill in treatment of allergic rhinitis. Chin J Chin Mater Med. 2014;39:2364–6. [Article in Chinese]. 25244776

[25] Lu YQ. Clinical effect observation of *Yu ping feng* granule in the adjuvant treatment for persistent allergic rhinitis. J Clin Exp Med. 2014;13:550–2. [Article in Chinese].

[26] Zhou WR, Zhang JX. Clinical effect observation of Chinese and western medicine integrative treatment for allergic rhinitis (36 cases). Jiangsu J Tradit Chin Med. 2010;42:42. [Article in Chinese].

[27] Huang J. Clinical analysis of Chinese and western medicine integrative treatment for allergic rhinitis. For all Health. 2014;8:17–8. [Article in Chinese].

[28] Liu GR (2012). Clinical effect observation of modified *Yu ping feng san* in treating perennial allergic rhinitis with deficiency of the lung-qi.

[29] Chen L, Chen XW. Clinical effect observation of Cetirizine combined with *Yupingfeng san* in treating allergic rhinitis. World Chin Med. 2014;9:880–2. [Article in Chinese].

[30] Lin YG (2014). Clinical effect observation of modified *Yu ping feng san* in the treatment for perennial allergic rhinitis with the lung-qi deficiency cold, and the changes of interleukin-2 (IL-2) and interleukin-4(IL-4).

[31] Guan CL. Clinical effect observation of jade screen powder combined with budesonide nasal spray in the treatment for allergic rhinitis. Chin Med Pharm. 2013;3:71–2. [Article in Chinese].

[32] Chen ZJ, Wang WH, liu B, Zhao XY. Effects of Azelastine nasal spray plus *Yu ping feng* decoction on serum levels of IgE and peripheral eosinophils of allergic rhinitis patients. Mod J Integr Tradit Chin West Med. 2015;24(36):4040–2. [Article in Chinese].

[33] Ma JJ. Effects of *Yu ping feng* granule combined with mizolastine on inflammatory factors and immune function in patients with allergic rhinitis. J North Pharm. 2017;14(7):105–6. [Article in Chinese].

[34] Qiu HJ. Clinical effect of *Yu ping feng* granule combined with Ebastine in the treatment of allergic rhinitis. Inter Med Heal Gui News. 2017;23(4):533–5. [Article in Chinese].

[35] Zheng WW, Chen XH, Li HT, Luo YZ, Han JQ. Curative effect observation of *Yu ping feng* granule combined with cetirizine in the treatment of allergic rhinitis. Chin Mod Doc. 2017;55(10):73–9. [Article in Chinese].

[36] Society of Otorhinolaryngology, Chinese Medical Association; Editorial Board of Chinese Journal of Otorhinolaryngology. Criteria for diagnosis and therapeutic evaluation of allergic rhinitis (Revised in 1997, Haikou). Zhonghua Er Bi Yan Hou Za Zhi. 1998;33:134-5. [Article in Chinese].

[37] Editorial Board of Chinese Journal of Otorhinolaryngology Head and Neck Surgery, Chinese Otorhinolaryngology Society of Chinese Medical Association. Diagnostic and treatment principle for allergic rhinitis and a recommended scheme (2004, Lanzhou). Zhonghua Er Bi Yan Hou Tou Jing Wai Ke Za Zhi. 2005;40:166-7. [Article in Chinese]. 15952561

[38] Bi Qiu (allergic rhinitis). In: State Administration of Traditional Chinese Medicine of the People’s Republic of China. Standard of Diagnosis and Assessment of Treatment Effects of otolaryngological Conditions in Chinese Medicine. Nanjing; 1994. p. 24. [Article in Chinese].

[39] Chinese medicine clinical research guiding principles of new drugs for Bi Qiu (allergic rhinitis).In: Ministry of Health of the People’s Republic of China. Chinese medicine clinical research guiding principles of new drugs (the third version). Beijing, China; 1997. p. 170–2. [Article in Chinese].

[40] Kong CM, Zhao ZJ, Zhong XH (2013). Effects of *Gan lian Yu ping feng* powder on the antibody titers to infectious laryngotracheitis vaccine and some nonspecific immune indexes in chickens. Afr J Tradit Complement Altern Med.

[41] Makino T, Sasaki SY, Ito Y, Kano Y (2005). Pharmacological properties of traditional medicine (XXX): effects of Gyokuheifusan ([玉屏風散]) on murine antigen-specific antibody production. Biol Pharm Bull.

[42] Gao J, Li J, Shao X, Jin Y, Lü XW, Ge JF, Huang Y, Zhang L, Chen L (2009). Antiinflammatory and immunoregulatory effects of total glucosides of Yupingfeng powder. Chin Med J.

[43] Bousquet J, Bachert C, Canonica GW, Casale TB, Cruz AA, Lockey RJ, Zuberbier T (2009). Extended global allergy and asthma European network and world allergy organization and allergic rhinitis and its impact on asthma study group. Unmet needs in severe chronic upper airway disease (SCUAD). J Allergy ClinImmunol.

[44] Hellings PW, Fokkens WJ, Akdis C, Bachert C, Cingi C, Dietz de Loos D (2013). Uncontrolled allergic rhinitis and chronic rhinosinusitis: where do we stand today. Allergy.

[45] Dong H. Modified *Yu ping feng san* combined with Cetirizine treated allergic rhinitis (32 cases). Mod J Integr Tradit Chin West Med. 2012;21:984–985. [Article in Chinese].

[46] Li XY. Clinical effect observation of modified *Yu ping feng san* combined with Cetirizine treated allergic rhinitis (50 cases). Jilin Med J. 2013;34:460. [Article in Chinese].

[47] Zou LL, Zou SS, Xiong SW, Wu XZ. *Yu ping feng oral liquid* on the inhibition of influenza virus and its influence on the body’s immune function. J Chin Med Materials.1990;13:37–40. [Article in Chinese].

[48] *Yu ping feng san*. In: Ji YB. Compound Chinese Medicine Pharmacology and Application. Beijing, China: China Medical Science and Technology Press; 2005. p. 672–80. [Monograph in Chinese].

[49] Wen J, Zhu JM, Li J, Yuan GM, Xiang FJ. Experimental study of *Yu ping feng san* on allergic rhinitis in rat and guinea pig. Chin Tradit Patent Med. 2011;33:934–7. [Article in Chinese].

[50] Tong L, Liu JL, Wang JX, Sun LL, Song YL, Tian GY, He S, Gao YJ. Effect of *Yupingfeng* granule on cytokines of allergic rhinitis induced by OVA in rats. Chin J Chin Mater Med. 2016;41:728–30. [Article in Chinese]. 10.4268/cjcmm20160431 28871701

[51] Zhang ZL, Zhong L, Ling BD, Yuan MY. Effect of *Yu ping feng san* on activity of IL-6 and TNF-α in rats with allergic rhinitis. Chin Tradit Patent Med. 2014;36:1804–9. [Article in Chinese].

[52] Zhang ZL, Zhong L, Ling BD, Yuan MY. Experimental research of *Yu ping feng san* in regulating the activity of mast cells in the allergic rhinitis based on interleukin-3 and GM-CSF. J Chengdu Med College. 2014;9:392–6. [Article in Chinese].

[53] Xin HT, Hou LJ, Xu SH, Zhou XH. Immune pharmacological research progress of *Yu ping feng san*, Chin J Chin Mater Med. 1998;23:505–7. [Article in Chinese].

[54] Shen DD, Xie XJ, Zhu ZJ, Yu X, Liu HL, Wang HZ, Fan HW, Wang DW, Jiang GR, Hong M. Screening active components from *Yu-Ping-Feng-San* for regulating initiative key factors in allergic sensitization. PLoS One. 2014; 10.1371/journal.pone.0107279. 10.1371/journal.pone.0107279 PMC4157890 25198676

[55] Shen HH, Wang K, Li W, Ying YH, Gao GX, Li XB, Huang HQ (2008). AstragalusMembranaceus prevents airway hyperreactivity in mice related to Th2 response inhibition. J Ethnopharmacol.

[56] Lee YS, Han OK, Park CW, Yang CH, Jeon TW, Yoo WK, Kim SH, Kim HJ (2005). Pro-inflammatory cytokine gene expression and nitric oxide regulation of aqueous extracted Astragali radix in RAW 264.7 macrophage cells. J Ethnopharmacol.

[57] Khan S, Shin EM, Choi RJ, Jung YH, Kim J, Tosun A, Kim YS (2011). Suppression of LPS-induced inflammatory and NF-kappaB responses by anomalin in RAW 264.7 macrophages. J Cell Biochem.

[58] Dong HY, He LC, Huang M, Dong Y (2008). Anti-inflammatory components isolated from *AtractylodesmacrocephalaKoidz*. Nat Prod Res.

[59] Editorial Board of Chinese Journal of Otorhinolaryngology (Data sorting by Gu ZY). Criteria for diagnosis and therapeutic evaluation of allergic rhinitis. Zhonghua Er Bi Yan Hou Za Zhi. 1991;26:134. [Article in Chinese].

[60] Schulz KF, Altman DG, Moher D (2010). CONSORT 2010 statement: updated guidelines for reporting parallel group randomised trials. BMJ (Clinical research ed).

[61] Gagnier JJ, Boon H, Rochon P, Moher D, Barnes J, Bombardier C (2006). Reporting randomized, controlled trials of herbal interventions: an elaborated CONSORT statement. Ann Intern Med.

