# Supplementary material for: Potential effectiveness of Chinese herbal medicine Yu ping feng san for adult allergic rhinitis: a systematic review and meta-analysis of randomized controlled trials
Source: BMC Complement Altern Med. 2017 Nov 6;17:485. doi: 10.1186/s12906-017-1988-5 (PMC5674829; doi:10.1186/s12906-017-1988-5)
Supplement: Supplementary file 2 — Search Strategies. (DOCX 18 kb) [file 12906_2017_1988_MOESM2_ESM.docx]

**Search Strategies**

The search strategies for English Databases: #1 Allergic Rhinitis OR Hay Fever OR Pollinosis.

#2: Yu Ping Feng OR Yu Ping Feng San OR Yu Ping Feng Powder OR Yupingfeng OR Yupingfeng San OR Yupingfeng Powder OR Yu-ping-feng OR Yu-ping-feng-san OR Yu-ping-feng-powder OR Gyokuheifu-san OR GHS OR Jade Windscreen Powder OR Traditional Chinese Medicine OR Chinese Traditional Medicine OR Chinese Medicine herb OR Chinese Medicine Formula OR Chinese Herb OR Chinese Formula.

#3: Randomized Controlled Trial OR Randomized OR Randomly.

#4: #1 AND #2 AND #3.

The search strategies for Chinese Databases included Chinese Biomedicine (CBM), China Network Knowledge Infrastructure (CNKI), Wanfang Database and Chinese Scientific Journals Database (VIP) (Search by Using Simplified Chinese Character):

#1: Bian Ying Xing Bi Yan(Allergic Rhinitis) OR Guo Min Xing Bi Yan (Allergic Rhinitis) OR Bi Qiu (Allergic Rhinitis) OR Bian Tai Fan Ying Xing Bi Yan(Allergic Rhinitis) OR Bian Tai Xing Bi Yan (Allergic Rhinitis) OR Bian Tai Fan Ying Bi Yan (Allergic Rhinitis) OR Fan Ying Xing Bi Yan (Allergic Rhinitis) OR Ku Cao Re (Hay Fever) OR Hua Fen Re (Pollinosis) OR Hua Fen Zheng (Pollinosis) OR Hua Fen Bing (Pollinosis).

#2: Zhong Yi (Traditional Chinese Medicine) OR Zhong Xi Yi (Integrative medicine) OR Zhong Yi Liao Fa (Chinese Medicine Terapeutics) OR Bian Zheng Lun Zhi (syndrome diﬀerentiation and treatment) OR Bian Zheng (syndrome diﬀerentiation) OR Han Fang (Kampo) OR Zu Guo Yi Xue(Chinese Medicine) OR Chuan Tong Yi Xue (traditional medicine) OR Chuan Tong Zhi Liao (traditional treatment) OR Bu Chong Ti Dai Yi Xue (Complementary and alternative medicine) OR Zhong Guo Chuan Tong Yi Xue (traditional Chinese medicine) OR Min Zu Yi Yao (Ethnomedicine) OR Cao Yao (herbal medicine) OR Zhong Cao Yao (Chinese herb medicine) OR Zhong Yao Liao Fa (Chinese herb medicine therapeutics) OR Zhong Xi Yao (Chinese and western medicine) OR Zhong Cheng Yao(Chinese patent medicine) OR Yu Ping Feng OR Yu Ping Feng San (Yu ping feng powder) OR Yu Ping.

#3: Lin Chuang Guan Cha (clinical observation) OR Lin Chuang Shi Yan (clinical trial) OR Lin Chuang Yan Jiu(clinical research) OR Liao Xiao Yan Jiu (effectiveness research) OR Liao Xiao Ping Jia (effectiveness evaluation) OR Qian Zhan Xing (prospective) OR Dui Zhao (control) OR Sui Ji (random) Duo Zhong Xin (multiple centres).

#4: #1 AND #2 AND #3.
